# Metagenomics: a path to understanding the gut microbiome

**DOI:** 10.1007/s00335-021-09889-x

**Published:** 2021-07-14

**Authors:** Sandi Yen, Jethro S. Johnson

**Affiliations:** Oxford Centre for Microbiome Studies, Kennedy Institute of Rheumatology, University of Oxford, Roosevelt Drive, Headington, Oxford, OX3 7FY UK

## Abstract

The gut microbiome is a major determinant of host health, yet it is only in the last 2 decades that the advent of next-generation sequencing has enabled it to be studied at a genomic level. Shotgun sequencing is beginning to provide insight into the prokaryotic as well as eukaryotic and viral components of the gut community, revealing not just their taxonomy, but also the functions encoded by their collective metagenome. This revolution in understanding is being driven by continued development of sequencing technologies and in consequence necessitates reciprocal development of computational approaches that can adapt to the evolving nature of sequence datasets. In this review, we provide an overview of current bioinformatic strategies for handling metagenomic sequence data and discuss their strengths and limitations. We then go on to discuss key technological developments that have the potential to once again revolutionise the way we are able to view and hence understand the microbiome.

## Introduction

### The holobiont

The ability to sequence, assemble, and analyse whole genomes has sparked a genomic revolution that began with the completion of the human genome and continues today (Choudhury et al. 2020; Lander et al. 2001; Weinstock 2007). However, it is increasingly apparent that the human genome does not operate in isolation, rather it is part of a holobiont; a co-existing and co-evolving collection of host and microbial genomes that encompasses not just all three domains of life, but also viruses (Zilber-Rosenberg and Rosenberg 2008).

Applying the same high-throughput sequencing technologies that revolutionised human genomics to the microorganisms “that literally share our body space” (Lederberg and McCray 2001) has resulted in a genomic revolution of its own. While characterising bacteria on the basis of single genes, such as the 16S ribosomal RNA gene, is a long-established technique (Fox et al. 1977) that has been successfully carried over into the modern genomic era, more recent shotgun sequencing approaches have proved revolutionary because they enable the capture of entire microbial genomes. In the context of this review, the collective term for these genomes is the metagenome (Handelsman et al. 1998), which may be thought of as the microbial genomic contribution to the mammalian holobiont (Bordenstein and Theis 2015).

### Technology is the gatekeeper of the microbiome

As genomes encode functional potential at the cellular, organismal, and holobiont level, accessing the metagenome through metagenomic whole genome shotgun sequencing (mWGS) holds the key to understanding the multifaceted roles that microorganisms play in determining host health.

Of course, taking a purely sequence-based perspective on the mammalian microbiome belies the generations of dedicated microbiological research that are the foundation of this field. However, culture-based approaches that are the cornerstone of microbiology have historically been a bottleneck in holobiont studies, due to the challenges associated with isolating, culturing, and subsequently studying the sheer number and diversity of microorganisms present in the human microbiome (Lloyd-Price et al. 2016). Sequence-based approaches indicate that between 150 and 200 unique bacterial species reside in a healthy human gut (Qin et al. 2010; Yang et al. 2020), many of which have, until recently, been considered unculturable (Walker et al. 2014). The number and diversity of other key members of the microbiome, such as viruses and fungi are more difficult to predict (Hallen-Adams and Suhr 2017; Sutton and Hill 2019). In consequence, while culturing remains an essential and evolving part of microbiome research (Browne et al. 2016; Rajilić-Stojanović and de Vos 2014; Walker et al. 2014), it is advanced in sequencing technologies that have resulted in a paradigm shift, leading to new language (Lederberg and McCray 2001), as well as new perspectives on human health (Inkpen 2019).

For microbiome studies, the next-generation sequencing (NGS) technology that has done most to bring about this paradigm shift is the massively parallel sequencing enabled by second-generation sequencing platforms. This has allowed massively parallel detection, quantification and, in the case of metagenomics, characterisation of thousands of microbial taxa within a single sample. While early sequencers, such as the Roche GS-FLX, were capable of producing 4–6 million bases of sequence per run, current state-of-the-art platforms such as the Illumina NovaSeq are able to produce up to six terabases. Such advancement has decreased the cost per base of sequencing (reviewed in Levy and Myers 2016) to a point where it is widely accessible and, in doing so, it has provided the lens through which the metagenome can be assessed.

Subsequent arrival of third-generation technologies, such as nanopore sequencing (Oxford Nanopore Technologies) and single-molecule real-time sequencing (Pacific Biosciences), has coupled massively parallel sequencing with the ability to produce long reads (typically tens of thousands of bases per read). For microbiome research, long reads have meant a greater ability to identify the taxonomic origin of reads and hence better understand the composition of microbial communities. They have also improved the ability to assemble and annotate individual genes and genomes, leading to improved functional characterisation.

Although advances in sequencing technologies are fundamental to metagenomics, they are not the only enabling technology that should be credited with the recent microbiome revolution. As with other genomic research fields, concurrent advancement in computational capacity, leading to continuous reduction in computing and storage costs has also been critical in order to keep pace with the ever-increasing amounts of microbiome sequence data produced.

This close association between genomic sequencing and computational capacity has been recognised since the early days of the human genome project. However, we feel there is also a third revolution that deserves acknowledgement for enabling recent advances in metagenomics. This is the open-source model for software development. There is the widely acknowledged benefit of open collaboration for overcoming computational challenges associated with the ever-growing scale and progressively changing nature of NGS data. Furthermore, there is the continued effort of computer scientists to put bioinformatic tools into the hands of biologists who would not otherwise be able to develop them. While bioinformatic analysis of metagenomic sequence data arguably still remains a bottleneck (Scholz et al. 2012), efforts to make rapidly evolving software freely available and accessible to the users best positioned to interpret their output have been instrumental in expanding our understanding of the metagenome and host–microbiome interaction.

The fact that bioinformatics has moved beyond the preserve of specialists, towards being a ubiquitous presence within most research groups is testament to the success of this movement. The purpose of this review is to give genome biologists unfamiliar with the microbiome an introduction to current computational approaches for handling mWGS data, with a specific focus on exemplary methods, the challenges they overcome, and the insights they can yield.

### The essential challenges of mWGS data: ‘what’s there?’ and ‘what does it do?’

Assuming the ultimate goal of most human and mouse microbiome studies is to understand the mechanisms by which microbes influence host health, there are, broadly speaking, two approaches that can be taken with mWGS data. The first is to ask ‘what’s there?’. Establishing the taxonomy of microbes present in a sample and quantifying their relative abundance enables correlative association between taxa (or community-level characteristics such as diversity) and host traits of interest. The second approach is to ask ‘what does it do?’. Detecting and quantifying genes present in the metagenome and inferring their function enables insight into higher-order biological processes encoded by the metagenome.

One important caveat to the second approach is that the presence of a gene within a metagenome does not necessarily mean that it is expressed. Metatranscriptomics is an alternative, and sometimes complementary approach to mWGS that has the potential to directly quantify gene expression within metagenomic samples (reviewed by Zhang et al. 2021). However, applying RNA-sequencing to the microbiome presents its own unique challenges and remains a comparatively underutilised approach that is beyond the scope of this review.

While mWGS data have the potential to provide information on both taxonomy and function, what is useful depends on the context of a particular study. For example, rapid detection of pathogens relies primarily on being able to resolve microbial taxa. A recent study by Gu et al. (2021) used mWGS of cell-free DNA to detect multiple known bacterial and fungal pathogens in human body fluids such as bronchoalveolar lavage, pleural fluid, and cerebrospinal fluid. In several cases, sequence-based detection was sufficient to identify causative microbes that were missed by more conventional techniques.

On the other hand, taxonomic profiling may not always be informative in metagenome studies where specific functions within the holobiont may be performed by multiple different taxa (i.e. functional redundancy), whose occurrence may vary between individuals, populations, or geographic regions. Early metagenomic characterisations of the human gut microbiome observed that while taxonomic composition varied between individuals, encoded functional potential tended to be much more conserved (Huttenhower et al. 2012; Turnbaugh et al. 2009). Furthermore, it is also possible for genomic content to vary substantially between strains of the same species (e.g. acquired antibiotic resistance through horizontal gene transfer), meaning that in some cases taxonomy also may underrepresent functional diversity.

The requirement to identify taxonomic abundance and/or functional potential means that many bioinformatic approaches have been developed to address these specific challenges using sequence data. While this review does not attempt an exhaustive comparison of all available software, we nonetheless argue that functional redundancy in bioinformatic approaches is as important as functional redundancy in the gut microbiome itself. It engenders both resilience and the ability to adapt to changes in the technology-enabled sequencing landscape.

Two challenges, however, are fundamental to both taxonomic and functional characterisation of the gut metagenome. The first, as discussed, is the continued increase in the size of sequence datasets. The second is what is increasingly referred to as the ‘dark matter’ of the metagenome. In spite of massive efforts to sequence and analyse the human and mouse metagenomes, both remain incompletely characterised, largely due to their sheer size and complexity. The human microbiome has been estimated to contain in excess of 30 million microbial genes (Lloyd-Price et al. 2016) across all body sites, and recent meta-analyses have identified 22 million and 4.6 million unique genes in human and mouse guts, respectively (Lesker et al. 2020; Tierney et al. 2019). In the case of humans, inter-individual variability is also a key characteristic, with a meta-analysis of 2182 gut metagenomes by Tierney et al. (2019) indicating that up to half of detected microbial genes were unique to a single individual.

## Metagenomic approaches for taxonomic characterisation of the gut microbiome

### Reference databases for taxonomic classification

The growth in volume and diversity of published sequence data is reflected in the number of reference databases that exist to curate these data and make them publicly available (see for example www.oxfordjournals.org/our_journals/nar/database/cap/), with new compendiums appearing yearly to meet evolving biological interests (Rigden and Fernández 2021). Many of the databases that underpin metagenomic analysis are not specific for the purpose. They tend to be joint genome curation projects involving multiple major funding bodies in order to sustain the hands-on maintenance required to keep information current.

Metagenome databases suitable for taxonomic classification primarily revolve around archiving, mining, and annotation of individually sequenced bacterial genomes. The National Center for Biotechnology Information (NCBI), European Nucleotide Archive, and DNA Data Bank of Japan, together make up the International Nucleotide Sequence Database Collaboration (INSDC www.insdc.org), which serves as the primary global repository of genome sequences from all domains of life, as well as viruses. The US Department of Energy Joint Genome Institute (JGI) also hosts several bioinformatic resources to centralise access to genomic and metagenomic data. JGI’s Genome Online Database (GOLD, Mukherjee et al. 2021) and Integrated Microbial Genomes and Microbiomes (IMG/M, Chen et al. 2019) are complementary tools that facilitate the classification of metagenomic data. The former is a registry for genome and metagenome projects and ensures complete documentation of metadata associated with each project. The latter specialises in access, annotation, and analysis of microbial genome and metagenomes. IMG/M connects external taxonomic and functional annotation databases via several bioinformatics pipelines for comparative analyses.

Such integration is a key feature of metagenomic reference databases, as extensive interrelation and sharing of genomes allows for varying degrees of curation within different resources. For example, GenBank (Sayers et al. 2020) is the INSDC-supported database from which the NCBI RefSeq database (Haft et al. 2018; O'Leary et al. 2015) is curated to provide a non-redundant set of high-quality genomes with established taxonomy and comprehensive annotations. The Genome Taxonomy Database Base (GTDB, Parks et al. 2020, 2018) and proGenomes (Mende et al. 2017) are examples of two subsequent databases built upon these NCBI resources, which further seek to standardise taxonomic annotation of bacterial and archaeal genomes. Both GTDB and proGenomes assign genomes to species clusters and curate non-redundant sets of genomes for different taxa. Curating standardised databases streamlines database query results based on taxonomy, which provides marginal advantages due to the size and scale of these metagenome databases. For example, GTDB and proGenomes have ~ 150 000 and ~ 84 000, bacterial and genomes, respectively, which span tens of thousands of species clusters. Efficient data mining, effective database management and integration, and scalable comparative genomic analyses are major priorities in metagenome reference databases.

#### Non-bacterial reference databases

While there has been an historical emphasis on bacterial members of microbiome, there are a growing number of reference databases that specifically support study of non-bacterial taxa. This includes fungi (mycobiome, Lai et al. 2018), viruses (virome, Carding et al. 2017), archaea (archaeome, Moissl-Eichinger et al. 2018), nematodes (Harris et al. 2019), and eukaryotic parasites (Dheilly et al. 2017). Metagenome resources for more targeted questions, such as pathogen genomics, are also available. For example, the Virus Pathogen Resource (ViPR, Pickett et al. 2012), and Eukaryotic Pathogen, Vector and Host Informatics Resource (VEuPathDB, Aurrecoechea et al. 2010). Given their more clinical focus such databases frequently also incorporate sample or clinical metadata relevant to each submission, either internally, or linked in related database resources such as Clinical Epidemiology Database Resources (ClinEpiDB, Ruhamyankaka et al. 2020).

#### Current limitations of reference databases

In spite of huge efforts to curate high-quality references and maximise their accessibility, a fundamental limitation of existing databases is that they are missing the ‘dark matter’ of the global metagenome. While gauging the extent of this problem is not trivial, it has been attempted. For example, Zhang et al. (2020) matched all unique taxa identified by the Earth Microbiome Project (EMP) using 16S gene sequencing to all available reference genomes in the RefSeq database. A median of 62% of EMP taxa present in host-associated microbiomes could be matched to an existing RefSeq genome at a threshold of 97% similarity. This enables the crude estimate that up to ~ 40% of mammalian holobiont bacterial species may be missing from reference genome databases. It is less clear what proportion of eukaryotic, viral or archaeal data may be missing. However, similar issues are likely to exist, particularly, as databases specialised in non-bacterial organisms tend to be comparatively small. For example, eukaryotic databases such as WormBase and VEuPathDB have in the range of 1–200 unique species for each family.

A second, related issue is that microbial reference databases tend to show distinct taxonomic bias. The historical need for microbial reference genomes to have come from organisms successfully isolated from environmental samples means this bias is weighted towards those taxa that can be successfully cultured (Browne et al. 2016). It also reflects uneven distribution of research effort towards organisms of particular interest, such as model organisms or important pathogens, as well as a bias towards microbes associated with human populations that have been disproportionately studied (Pasolli et al. 2019).

An unfortunate consequence of both these limitations is that potentially important biological associations may be lost in the proportion of mWGS reads that are returned as ‘unclassified’ in metagenomic studies. This risk is greater when considering microbial clades that are comparatively understudied, or human populations that are historically not well represented. While these limitations do not preclude use of reference databases as an essential means of taxonomic classification, they need to be accounted for when interpreting metagenomic analyses and represent a challenge that needs to be overcome if we are to improve our understanding of the microbiome contribution to host health.

### Matching sequences to reference databases

#### Sequence Pre-processing

Quality control is a fundamental upstream process in all sequenced-based analysis, involving steps such as the removal of likely PCR duplicates, removal of sequencing adapters, trimming of low-quality bases from reads, and the masking or removal of low complexity regions. One fundamental pre-processing step is the removal of sequences originating from contaminant-DNA (i.e. DNA that is not the focus of the study). While this is an important consideration in single genome studies (Goig et al. 2020), it represents a particular challenge in mWGS, where host DNA likely to be highly prevalent in samples and hence constitute a significant fraction of the sequenced reads.

Removal of host reads is a vital precursor for both taxonomic and functional characterisation of the gut microbiome. Failure to do so may confound taxonomic estimates through misassignment of host reads to microbial taxa. This may be of particular importance for virome analysis due to the presence of endogenous retroviruses, which are estimated to make up approximately 8–10% of the human and mouse genomes (Lander et al. 2001). Failure to remove host reads may also result in chimeric assemblies, which can in turn lead to annotation of spurious proteins that can confound both taxonomic and functional analysis (Breitwieser et al. 2019).

Removal of host reads is fundamentally a case of matching sequences to a reference database (albeit one containing only the host reference genome), as such the approaches reviewed below (local alignment, alignment-free sequence classification) are applicable to this problem.

#### Local alignment

The earliest published attempts to shotgun sequence the human gut metagenome employed de novo sequence assembly, before matching reassembled genes and genomic fragments to in-house protein reference databases using BLASTP (Gill et al. 2006; Kurokawa et al. 2007). Since these landmark studies, local alignment of query sequences to reference databases with previously ascribed taxonomy has remained a cornerstone of metagenomic characterisation.

The recent massive growth in the size of both query and reference datasets has meant that early alignment tools such as BLAST are no longer computationally efficient for the analysis of metagenomic sequence data. Fast and accurate characterisation of microbial sequences remains a research priority, particularly, in large-scale disease studies where microbiome datasets may extend to thousands of samples, each with tens of millions of reads (e.g. Lloyd-Price et al. 2019), or in clinical studies, where speed and accuracy of diagnosis are likely to be critical (Chiu and Miller 2019).

As with other genomics fields, metagenomics has benefited from the fact that the continuous advancement of technologies has inspired reciprocal development of many alignment tools optimised to cope with both the scale and features of sequence data produced on NGS platforms (Fonseca et al. 2012). A key element that sets apart tools suited for metagenomic analysis is their ability to efficiently index reference genomes so that they can be accessed and searched with great speed. Indexing is a particular challenge for metagenomic analysis, where reference sequence databases may be an order of magnitude larger than the databases required to represent single mammalian genomes.

For second-generation mWGS data, two of the most widely adopted tools for metagenomic sequence alignment are Bowtie (Langmead and Salzberg 2012; Langmead et al. 2009) and BWA (Li and Durbin 2009). Both use the FM-index in conjunction with the Burrows–Wheeler Transform (reviewed in Canzar and Salzberg 2017) to efficiently index and compress reference sequences for rapid searching. While both approaches achieve significant improvements in alignment speed, they also still rely on heuristic ‘seed-and-extend’ approaches (reviewed in Ahmed et al. 2016), and are hence not guaranteed to find optimal alignments for all query sequences. Such heuristic assumptions are likely to have little practical impact in situations where the resulting alignment accuracy is sufficient to correctly assign reads to divergent taxa (Al-Ghalith and Knights 2020). However, as microbiome studies move towards an increasing emphasis on the ability to accurately discriminate between closely related strains, recently developed rapid, heuristic-free short-read aligners such as BURST (Al-Ghalith and Knights 2020) are likely to become increasingly important in microbiome studies.

Current state-of-the art short-read alignment approaches, such as BWA, have also been adapted for alignment of the longer reads typically produced by third-generation sequencing platforms (Li 2013). However, aligners such as BLASR (Chaisson and Tesler 2012) and Minimap2 (Li 2018) have also come to the fore, having been specifically designed to overcome the sequencing errors encountered on these platforms. More recently, for ONT, these approaches have been further improved by the ability to predict and model the structure of errors inherent to nanopore sequencing (Joshi et al. 2020).

#### Alignment-free sequence classification

The speed at which reads can be aligned using state-of-the-art short and long-read aligners means such approaches remain viable for searching increasingly large numbers of query reads against ever-growing references databases. However, when the exact location of a specific read within a reference genome is not important, as is the case when the primary goal is to estimate taxonomic origin, precise alignment represents an unnecessary computational cost.

The relative computational efficiency of matching exact kmers, rather than long, potentially ambiguous reads, means matching query sequences to reference databases based on the overall similarity their respective kmer compositions is an extremely rapid way to achieve alignment-free classification of metagenomic sequences (Ren et al. 2018). For example, Kraken—one of the most widely used metagenomic taxonomic profiling tools—divides reference genomes (by default, derived from RefSeq) into kmers, then assigns each unique kmer to the lowest taxonomic rank that represents all the genomes in which it can be found (a so-called lowest common ancestor (LCA) approach). Kmers from query sequences can then be matched to this taxonomy. The taxonomic origin of a complete read can then be inferred from the distribution of its constituent kmers within the underlying taxonomic tree.

#### Resolving ambiguous classification

Not only it is possible to use the LCA to infer the taxonomy of a read based on the classification of its constituent kmers, it is also possible to use this approach to classify aligned reads that ambiguously align to multiple references. For example, MEGAN (Huson et al. 2007) provided an early implementation of this method to assign taxonomy to locally aligned microbial sequences, which has recently been adapted to work with third-generation sequence data (Huson et al. 2018).

While LCA strategies offer a robust approach to taxonomic classification, a recent study has suggested that trends in the growth of underlying reference databases potentially limit their ability to classify sequences at species level, or strain level (Nasko et al. 2018). Specifically, the authors noted the that recent massive expansion in the number of bacterial genomes in RefSeq has resulted in rapid increase in the number of species accessions to databases, but little expansion in the number of genera represented. The increasing species-to-genera ratio (and hence increasing number of genomes displaying a high degree of sequence homology at species level), leads to a reduced ability for LCA approaches accurately assign taxonomy at species level. This observation has led to a call for continued development of such methods to maximise taxonomic resolution while minimising the risk of false-positives.

One potential solution to this problem is to probabilistically reassign ambiguously classified reads to their most likely taxon of origin. Such an approach has been implemented for aligned reads in the PathoID module within the PathoScope pipeline (Hong et al. 2014). An analogous approach has also been developed for Kraken (Lu et al. 2017), which, rather than reassigning individuals reads, provides species-level abundance estimates based on LCA read assignments. Such approaches are likely to become increasingly relevant due to both the growing interest in understanding the microbiome at high taxonomic resolution, as well as the increasing levels of sequence homology within taxonomic reference databases.

#### Refining reference databases to reduce search space

Increasing the speed with which query sequences can be matched to sequences in reference database with known taxonomy is one way to overcome challenges inherent in taxonomic profiling of metagenomic sequence data. A second is to curate reference databases to remove redundant information that either does not discriminate between taxa, unnecessarily lengthens search times, or both.

As with read alignment and profiling, multiple approaches have been developed that exploit different characteristics of the metagenome in order to design computationally efficient references. One such approach is to leverage the pan-genome concept, which encompasses the fact that bacterial strains of the same species consist of a core genome (present in all strains) and a dispensable genome (consisting of those genomic regions that may be present in some, but not all strains, Medini et al. 2005). The pan-genome is therefore the combination of the core and dispensable genomes for a species. This concept becomes increasingly relevant as microbial reference databases move from having one representative genome for each species, towards multiple and sometimes thousands of different strains. Zhou et al. (2018) exploited this concept by creating a reference database consisting solely of species pan-genomes. Resulting references were 2–20 times smaller in size (bp) than the total size of contributing strain genomes. Furthermore, this pan-genome database resulted in improved rates of read classification over databases including only a single representative genome for each species.

A second approach to minimising the size of a reference database, while retaining its ability to taxonomically classify query sequences is to retain only discriminatory genes that are unique to a single species (Segata et al. 2012). Metaphlan is based on this concept and uses local alignment with Bowtie2 to match query mWGS reads to gene families that are selected to be both present in a species core genome, and unique to that species (Beghini et al. 2020). Taking this approach Metaphlan3 is able to efficiently represent over 13,000 microbial species with a reference database of approximately 1.1 million marker genes. Such minimal reference databases, actually result in very few reads in being successfully aligned from query metagenomic datasets, but they are nonetheless sufficient to provide accurate taxonomic profiling of complex microbial communities. Furthermore, the lightweight design of such discriminatory gene databases means that they are likely to scale efficiently with the increasingly large amounts of data processed in single studies, when compared to approaches that depend on de novo assembly of metagenomes (Segata 2018).

The concept of restricting databases to discriminatory markers is not limited to microbial gene sets. Tu et al. (2014) introduced a method for identifying and selecting unique, discriminatory regions from reference genomes (termed genome specific markers—GSMs). They subsequently matched query reads to these GSMs using BLAST-like approaches. More recently, CLARK (Ounit and Lonardi 2016; Ounit et al. 2015) is a kmer-based classifier, comparable to Kraken, that not only seeks to exploit the speed of kmer-based searching, but also to reduce the size of the reference database by storing and searching only discriminatory kmers. This has the advantage of minimising the amount of information that needs to be stored to quickly and accurately discriminate reads. However, it also means that the taxonomic level at which reads are to be classified (i.e. at which a single kmer is unique to a clade) needs to be specified prior to building a reference index and hence that an LCA approach to read classification cannot be taken.

### Beyond reference databases: the dark matter of the metagenome

As discussed, a fundamental problem of assigning taxonomy to metagenomic sequences by matching them to reference databases is that these databases are almost certainly incomplete. While LCA approaches, such as Kraken, may be able to classify reads originating from unknown microbes at higher taxonomic levels, detecting and quantifying and characterising these microbes at low taxonomic resolution remains a major challenge.

#### Reference-extended approaches

Discriminatory marker gene databases, such as those curated by MetaPhlan, allow users to quantify the unclassified proportion of reads in an mWGS dataset. However, they provide no additional insight into reads originating from taxa missing from the reference genome databases from which they are derived. Other approaches based on sets of universal marker genes offer a potential solution to this problem. In mOTU (Milanese et al. 2019; Sunagawa et al. 2013), the authors curate a database of single-copy marker genes identified as present in all sequenced microbial genomes. They then use a hidden Markov model (HMM)-based approach to generate a profile for each marker gene, based on its sequence properties across known reference genomes (reviewed in Eddy 1996). Such profiles can be used to search for all homologs of each marker gene within de novo assemblies of metagenomic samples. All detected copies of each marker gene are clustered in a step analogous to the generation of operational taxonomic units (OTUs) from 16S gene sequence data. The relative abundance of each ‘meta-OTU’ (mOTU) is then determined, and mOTUs originating from the same genome are identified based on correlation in their relative abundance across samples. While this innovative approach relies on the computationally challenging step of de novo metagenome assembly, the use of HMM profiles enables detection of marker gene homologs that may be absent from existing genome reference databases, thereby enabling what the authors refer to as a reference-extended community profiling. In a recent study, the authors concluded that more than half the mOTU species detected in 1693 human gut samples were absent from the proGenomes reference database (Milanese et al. 2019).

#### Sequence-based community profiling

While reference-extended approaches offer the ability to define and quantify previously uncharacterised taxa, it is also possible to compare metagenomes entirely on the basis of sequence composition, without the need to define taxonomic units. The utility of such an approach reflects the fact that changes in the composition of the gut metagenome, such as dysbiosis, are often characteristic of disease states. Tracking such changes is therefore informative, in spite of the fact it contributes little to our understanding the mechanisms by which microbes impact host health (Olesen and Alm 2016). With this goal in mind Kmer-based approaches once again represent a computationally efficient method by which to compare the composition of metagenomic samples. MASH (Ondov et al. 2016) is an implementation of the MinHASH algorithm (reviewed in Rowe 2019), which provides an extremely fast method for approximating the proportion of kmers shared between two metagenomes. The utility of this type of approach has since been extended to account for the relative abundance of kmers when assessing samples, and to enable signatures to be searched as well as compared (Pierce et al. 2019).

#### Genome-Resolved Metagenomes

The ability to re-assemble complete, high-quality microbial genomes from shotgun sequence data is arguably the apotheosis of computational metagenomic analysis as it obviates the need to isolate and culture in order to understand the genomic potential of individual organisms. Genomes that may never be cultured can be retrieved, their phylogeny can be established and taxonomy inferred (Almeida et al. 2019; Almeida et al. 2020), and their functions predicted through genome annotation. Ultimately, these genomes can be added to public databases (Almeida et al. 2020; Mukherjee et al. 2021), leading to the improved performance of other, reference-dependent analysis tools (Milanese et al. 2019).

Full or partially assembled genomes derived from mWGS data are now commonly referred to as metagenome-assembled genomes (MAGs). They were first generated from shotgun sequencing of biofilms by Tyson et al. (2004), who assembled 103,462 Sanger reads (76.2 Mb), then binned the resulting contigs into genomes based on a combination of their coverage and GC content. In another landmark study, Nielsen et al. (2014) analysed 396 human stool samples (23.2 billion reads, 4.5 Gb) as part of the MetaHIT consortium. They used a canopy clustering approach to enable the rapid binning of assembled microbial genes based on their co-abundance across samples. This resulted in detection of 784 metagenomic species (defined as bins with > 700 genes) and also demonstrated the potential for MAG approaches to identify bacteriophage. More recently, massive efforts have been made to reconstruct genomes from publicly available metagenomic sequence datasets Nayfach et al. (2019), Pasolli et al. (2019), and Almeida et al. (2019) analysed 3810, 9428, and 11,850 metagenome samples, respectively, which have collectively contributed to a novel reference catalogue of 204,938 MAGs (Almeida et al. 2020).

Fundamental steps for generating MAGs are the production of high-quality de novo assemblies from mWGS reads (through use of tools such as metaSPAdes, Nurk et al. 2017, and MEGAHIT, Li et al. 2015, reviewed in Ayling et al. 2020) followed by the accurate binning of contigs originating from the same genome. The latter step is frequently performed by comparing the coverage of contigs, as well as genome-level sequence properties such as CG content or tetranucleotide frequency (reviewed in Kang et al. 2016). A widely used exemplar approach for binning is MetaBAT (Kang et al. 2015, 2019), which employs pairwise comparisons of contigs based on abundance and TNF frequencies, followed by a graph-based clustering approach (Kang et al. 2019) to identify MAGs from one or more samples.

The potential for MAGs to extend knowledge of the metagenome beyond reference databases is well illustrated by recent large-scale studies. For example, Pasolli et al. (2019) used MASH to establish pairwise genetic distances between 154,723 MAGS from different human body sites and 80,990 bacterial genomes from reference databases. Clustering these genomes at a 5% threshold resulted in an estimated 4,930 species, with 3,796 (77%) of these species clusters containing no previously known reference genome. Notably, novel MAGs are found at much greater frequency in non-westernised gut microbiomes (Nayfach et al. 2019; Pasolli et al. 2019), supporting the observation that genomic biases exist as much for the microbial proportion of the holobiont as they do for the host (Almeida et al. 2019; Choudhury et al. 2020). Further efforts to improve metagenomic discovery of microbial species are therefore likely to particularly benefit understanding the microbiome contribution to host health in these populations.

Another area in which the ability to fully resolve genomes from metagenomes offers great potential is the detection and characterisation of the non-bacterial component of the gut microbiome. This is well illustrated by recent studies of crAssphage, where mining publicly available metagenome assemblies for circular metagenome-assembled genomes (cMAGs) led to the discovery of 596 crAssphage genomes, which could be clustered into approximately 221 viral ‘species’ (Yutin et al. 2021). These viruses have subsequently been shown to be globally present in the human gut, where they dominate the gut virome and to have close biological links with the genus Bacteroides (Edwards et al. 2019; Yutin et al. 2021), which is itself a keystone species within the gut ecosystem. Recent success in the detection and characterisation of crAssphage is undoubtedly aided by their high relative abundance compared to other viral clades. Nonetheless, comparable metagenomic discovery of other microbes, from viruses to eukaryotes, remains a prospect for future studies.

While the potential for genome-resolved metagenomics is great, recent reviews have highlighted the challenges in this field, and in particular, the difficulties of producing high-quality assemblies of complex microbial communities where strain-level divergence may be important (Chen et al. 2020). The appearance of incompletely resolved, composite MAGs in public databases has already been reported (Shaiber and Eren 2019), and ensuring accurate and high-quality genome discovery remains a key bioinformatic challenge for this emerging field (Bowers et al. 2017).

## Metagenomics for functional characterisation of the gut microbiome

### Reference databases for assigning function to genes

The ability to map genes to functions and ultimately to higher-level biological processes that reflect mechanisms of host–microbiome interaction is a crucial step in metagenomic analysis. While a similar challenge exists for all genome-level research, it is arguably greater for metagenomics, where the proportion of uncultured and undescribed microbial genomes reflects a comparable proportion of novel microbial genes whose function is also unknown (Tierney et al. 2019).

One of the largest resources for metagenome annotation and functional interpretation is the Kyoto Encyclopaedia of Genes and Genomes (KEGG), which curates functional information at both a genomic and systems level (Kanehisa et al. 2017). At the genomic level, genes in the KEGG GENES database are principally obtained from the NCBI RefSeq and GenBank databases (inclusive of eukaryotes, prokaryotes, and viruses). Genes are subsequently clustered based on sequence similarity to form Kegg Orthology groups (KOs), which are functionally annotated. As of September 2020, the Kegg Orthology Database contained approximately 24,000 KOs, of which 82% were linked to experimentally characterised sequences (Kanehisa et al. 2021).

At a systems level, KEGG curates pathways representative of biological process and use of individual KOs as nodes within pathways enables the effective mapping of genes to systems. In metagenomic analysis, query sequences may be matched to KEGG genes, and thereby KOs. The explicit linking of KOs to pathways ultimately enables variations in microbial gene abundance to be related to variation in the genomic potential for specific biological processes.

MetaCyc is a database of almost 3000 literature-derived metabolic pathways, predominantly originating from prokaryotes (Caspi et al. 2020). Where available, protein sequence information for enzymes in pathways is sourced from the UniProt database. Meaning that, similar to KEGG, it is possible to match query metagenomic sequences to this database, typically via local alignment, to obtain estimates of the relative abundance of genes encoding specific pathways. Comparisons of the content of KEGG vs MetaCyc have found them to cover a comparable number of reactions (Altman et al. 2013), but evaluations are complicated by a lack of consistent terminologies and different pathway representations between the two resources.

KEGG, and to a lesser extent MetaCyc, are notable for their efforts to curate information at both the genomic and system level. However, other databases specifically dedicated to curating sequence-level information are also a valuable resource for functional annotation. The UniProt Knowledge Base (UniProtKB, Bateman et al. 2021) seeks to provide a complete database of all known protein sequences (the majority of which are derived from reference genomes), and link them to either experimentally verified or computationally predicted functions. Derived from UniProtKB, UniRef databases cluster protein sequences at 100, 90, or 50% identity to maintain non-redundant sequence catalogues (analogous to KOs), where each cluster is represented by a single seed sequence (Suzek et al. 2014). Annotations are largely consistent between clusters (Suzek et al. 2014), meaning alignment to UniRef seeds can be used as an effective method for assigning function to metagenome samples.

While some UniProt annotations may include formal pathway descriptions, the value of the database is further increased as a consequence of extensive efforts to match accessions to equivalent sequences, or higher-order information, in approximately 180 other databases (Huang et al. 2011). This includes both KEGG and MetaCyc, thereby allowing UniProt annotations to be indirectly mapped to curated pathways.

Another exemplary database that provides a sequence-level resource for functional annotation of metagenome samples is eggNOG (evolutionary genealogy of genes: Non-supervised Orthologous Groups, Huerta-Cepas et al. 2019). Protein sequences are again derived from selected representative eukaryote, prokaryote, and viral genomes. However, in this instance, they are grouped in a manner that distinguishes true orthologs (homologous sequences that have diverged due to speciation) from paralogs (homologous sequences diverged due to duplication) on the basis that the latter do not necessarily retain the same function. eggNOG does not manually curate functional annotations, but inherits broad functional categories from other ortholog databases (Galperin et al. 2015) and, like UniProt, provides extensive mapping to other functional annotation databases (http://eggnog5.embl.de/#/app/methods).

### Matching sequences to reference databases and biological functions

#### Metagenomic assembly and gene prediction

As first steps towards functional characterisation of mWGS samples, it is common to perform de novo metagenome assembly (a topic not covered here, but comprehensively reviewed in Ayling et al. 2020) followed by gene annotation. Metagenomic assemblies typically result in vast numbers of genomic fragments (contigs) rather than fully resolved genomes. While they are often far from complete, the assembly of short reads into such contigs enables full or partial gene sequences to be computationally predicted, extracted, and matched to reference databases containing genes of known function.

Gene prediction from metagenomic assemblies presents additional challenges when compared to gene prediction in individual genomes. Highly fragmented assemblies increase the likelihood of partial annotations. Additionally, common features that may unite (and hence help distinguish) genes within a single genome may not be shared across a metagenome. Well-established metagenome annotation tools such as MetaGeneMark (Zhu et al. 2010) and Prodigal (Hyatt et al. 2010) account for this, but potentially at the cost of their ability to make accurate novel gene predictions within the dark matter of the metagenome. In consequence, more recent tools seek to take advantage of the recent massive expansion in the number of annotated prokaryote genomes in order to improve the specificity of novel gene annotations from metagenome assemblies (Sommer and Salzberg 2021).

#### Functional annotation

As functional reference databases tend to curate homologous groups of genes, which are assumed to share a common function (for example, KEGG KOs or EggNOG orthologous groups), functional annotation can be performed by matching query sequences to groups, as well as alignment to individual reference sequences. HMM profiles are a powerful way to formally represent the sequence properties of a group (Eddy 1996). As such, they have the potential to improve metagenome functional annotation by matching of query sequences that bear strong similarity to the properties of a group, but limited similarity to each of its constituent members.

The curators of KEGG provide a dedicated server (KofamKOALA) that employs an HMM-based approach and can be used to generate KO classifications for translated metagenome annotations (Aramaki et al. 2020). Alternatively, they also support approaches that begin with BLAST-like alignment to the KEGG GENES database, before using the results to assign query sequences to a KO group (Kanehisa et al. 2016). The curators of eggNOG have similarly released a dedicated tool, eggNOG-mapper (Huerta-Cepas et al. 2017), which matches query sequences to eggNOG orthologous groups using pre-computed HMMs. However, for very large query datasets, classification speed may become an issue. This approach therefore also supports the use of DIAMOND (Buchfink et al. 2015); an extremely rapid translated aligner that can match queries against indexed seed sequences for each eggNOG orthologous group.

The annotation approaches discussed so far are primarily intended for the classification of complete or partial gene sequences. One alternative that allows direct quantification of function from unassembled reads is HUMAnN (HMP Unified Metabolic Analysis Network). The latest release version (HUMAnN3, Beghini et al. 2020) curates a custom database, combining protein sequences and functional annotations from UniProtKB with associated genome sequence and taxonomy information from GenBank. It subsequently performs a tiered search involving both nucleotide and translated alignment in order to maximise chances of successful read classification. One valuable consequence of this mapping approach is that, by retaining details of the original genomes to which reads map, HUMAnN is able to quantify the extent to which different taxa contribute to higher-order functions in databases such as MetaCyc and KEGG.

#### From genes to pathways

While the relative abundance of functionally annotated genes or orthologous groups within a metagenome may in some cases be informative, often higher-level biological processes are better represented by groups of genes (henceforth pathways) reflecting microbiome functions of interest (e.g. all genes contributing to the biosynthesis of secondary bile acids). Unfortunately, the quantification of such pathways in metagenomic samples is not trivial: individual genes may contribute to more than one pathway and the genes comprising a particular pathway may vary across taxa. In consequence, the number of tools that attempt to estimate a single measure of microbial pathway abundance from metagenomic data is limited. HUMAnN3 once again offers this utility through careful mapping of gene annotations to MetaCyc reactions and subsequently to higher-order pathways (Franzosa et al. 2018).

A common alternative to estimating a single measure of abundance for individual pathways is to treat the relative abundance of all genes (or orthologous groups if, for example, quantifying KEGG Orthologs) contributing to a pathway as a set, and subsequently to perform gene set analysis (GSA). GSA approaches in metagenomics are analogous to those used in other genomics fields (Huang et al. 2008). For example, it is possible to search for overrepresentation of a set within all genes identified as significantly differentially abundant between conditions, or alternatively search for enrichment of a set within a pre-defined ranking of all genes based on the extent and direction of their differential abundance between conditions.

GSA and pathway abundance measures offer invaluable insight into important biological processes encoded by mWGS data. However, the inherent difficulty in representing the millions of functionally and taxonomically diverse microbial genes as discrete pathways leads us to advise researchers to consider carefully the specific genes driving trends observed at higher functional levels.

### Challenges facing functional annotation

Extensive interrelation of sequence information across annotation databases is invaluable as a means of linking different complementary data sources. However, different routes to achieve the same information open up the potential for unappreciated biases due to differences in underlying bioinformatic algorithms. For example, HUManN3 aligned reads converted to estimates of KO relative abundance may not necessarily return the same results as quantifying the same KOs using the KEGG GhostKOALA server. Achieving the same ends via different means is not a problem per se, but the onus is on researchers to have a clear understanding of how they have derived the quantitative estimates of gene and pathway abundance they are working with.

Another issue facing functional annotation of metagenomic datasets is the dependency on manual curation for many of the higher-order biological pathways represented in databases. UniProtKB, KEGG, and MetaCyc are all to varying extents dependent on manual curation. Within UniProtKB the number of sequences with computationally predicted functions is growing at a greater rate than those with functions that have been manually annotated and reviewed. The contribution of MAG gene predictions to this database (UniProt Consortium 2019) is only likely to exacerbate this disparity. Again, the appearance of MAGs in databases used for functional annotation is not in itself problematic, but it rests on the assumption they are the result of high-quality assembly and highly stringent quality controls in order to maintain database integrity (Shaiber and Eren 2019). In databases such as KEGG and MetaCyc, the curation of pathways is heavily dependent on manual curation and pathway assignment is already restricted to a proportion of known genes: approximately 52% of KEGG genes are linked to a KO (Kanehisa et al. 2021), and approximately 50% of KOs are linked to pathways (Kanehisa and Sato 2019). In conclusion, ensuring functional annotation, in particular, curation of pathway-level information keeps pace with the growing number of newly discovered genes represents a major challenge for microbial informatics.

## Future perspectives: new technologies, new challenges

Current second and third-generation sequencing technologies have revolutionised our ability to dissect the composition and function of the gut microbiome. It is likely that future technological advancements will continue to provide similar step-changes in understanding and in doing so, present new bioinformatic challenges. One particular shortcoming of current metagenomic sequencing strategies is that the process of extracting and sequencing metagenomic material results in data that may not be easily mapped back to its original microenvironment. We end this review by briefly discussing exciting new technologies are emerging to address this limitation by preserving some of the contextual information of the metagenome.

### IgA-Seq

IgA is the primary antibody produced at gut mucosal surfaces, where it has been shown to bind different members of the gut microbiome with varying affinity. Identifying the IgA bound fraction of the microbiota can reveal taxa that are critical effectors of immune–microbiome interaction and likely to induce intestinal inflammation. This approach was first introduced by Palm et al. (2014) who used fluorescence-activated sorting (FACS) or magnetic-activated cell sorting (MACS) to determine IgA-binding, and subsequently 16S rRNA gene sequencing to determine taxonomy. It has since been successfully applied to examining host–microbiome interaction in IBD (Palm et al. 2014; Shapiro et al. 2021). For example, Shapiro et al. correlated IgA-coating with factors such as relative abundance, IBD treatment, and disease progression to describe the immunostimulatory effect of various taxa in different conditions. This study revealed that low IgA-coating in the genus *Oscillospira* was associated with worse disease progression. While early studies such as these coupled cell sorting with 16S sequencing, more recently it has been used in conjunction with shotgun sequencing (James et al. 2020).

### Spatially resolved microbiomes

The spatial environment surrounding microbiota imparts biologically important context for understanding metagenomic snapshots of the microbiome. This is particularly true in the gut where the microbiome composition has been shown to vary at fine scale between the gut lumen and the intestinal mucosal barrier (Duncan et al. 2020).

One recent approach for spatial resolution of microbial taxa is High Phylogenetic Resolution Fluorescent in situ Hybridization (HiPR-FISH, Shi et al. 2020). This method achieves taxon-specific tagging by using a probe that targets the 16S rRNA molecule and a secondary probe that is fluorescently labelled. The targeting probe is composed of an encoding sequence flanked by two non-identical read-out sequences, which complement the fluorescent secondary probe. A single taxon can be targeted with multiple probe tags to create a spectral barcode that is unique to that taxon. Pairs of fluorophores can be used to create a spectral barcode by concatenating the fluorescence emission spectra measured with five excitation lasers. This system can generate 1023 unique fluorophore pairs from 10 fluorophores. As such, probe sets can be designed to target multiple taxa in one assay, allowing for study of microbe–microbe interactions in multi-taxonomic bacterial communities. Imaging with single-cell resolution, the spectra emitted by each cell can be decoded with a machine-learning-based classifier to identify tagged cells and record their spatial coordinates.

Such quantitative spatial mapping creates opportunities to study microbe interactions with their surroundings. For example, the authors applied HiPR-FISH to spatially map microbiota in mouse colonic tissue and quantify spatial associations of bacterial taxa. The authors found that spatial association between pairs of bacteria, such as *Oscillibacter*-*Veillonella*, were disrupted with antibiotic treatment. In doing so, they demonstrated the potential for complex microbial communities to be studied at the single-cell resolution while addressing spatial orientation of microbiota.

### Single-cell metagenomics

Single-cell sequencing has revolutionised host genomics by providing the ability to describe discrete cell populations that would be lost to bulk transcriptomic approaches. The application of analogous approaches to the microbiome involves physically separating microbes into groups of either individual cells, or a few hundred cells—sometimes termed mini-metagenome. Several different technologies have been applied for this purpose, including flow-sorting cells, gel microdroplet cultivation (Fitzsimons et al. 2013), single droplet multiple displacement (Hosokawa et al. 2017), and microfluidic hydrogels (Chijiiwa et al. 2020). Chijiwa et al. (2020) used gel microfluidic hydrogels to compartmentalise individual cells. Cell DNA was then enzymatically lysed so that genomes could be amplified within each hydrogel, resulting in what they referred to as a Single-Amplified Genome (SAG). While SAGs had limited genome completeness, the authors were able to produce enough quality draft genomes to produce complete or composite genomes of SAG strains.

One notable advantage of this approach is that genomes of rare taxa can be assembled without having to dramatically increase sequencing depth. Additionally, SAGs only require tens of thousands of reads, allowing genomes to be multiplexed and mini-metagenomic sequencing to be performed on relatively modest platforms such as the Illumina MiSeq.

## Conclusions

Progress in gut microbiome research over the last two decades has been saltatory. In particular, the availability of affordable, second-generation sequencing technologies, coupled with easily accessible, open-source bioinformatic software has enabled researchers in many different disciplines to study the metagenome in order to understand the impact of the microbiome within their respective fields. This recent interest has led to an explosion in the amount of publicly available mWGS data.

While current bioinformatic approaches are able to keep pace with the demands of analysing increasingly large datasets, there is growing appreciation of the limitations of existing resources for the taxonomic classification and functional annotation of metagenomes. Bioinformatic approaches that enable the reconstruction of complete genomes from metagenomes offer a partial solution to this problem, enabling detailed characterisation of hitherto unknown microbial taxa. However, while such de novo approaches are able to effectively resolve microbial genes and genomes, the reliance on manual curation to collate and describe higher-order biological functions means there is still likely to be a significant bottleneck when it comes to extrapolating genome-level information to infer molecular the mechanisms that underpin host–microbiome interactions.

Continued advancement of novel and existing sequence-based technologies may contribute to solving these and other problems, enabling ever greater understanding of the microbe–microbe and host–microbe interactions that are relevant to host health.

## Data Availability

Not Applicable.
